# Outcomes of Continuous Enteral Vancomycin Infusion in Intensive Care Unit Patients: A Novel Treatment Modality for Severe Clostridium Difficile Colitis

**DOI:** 10.7759/cureus.22872

**Published:** 2022-03-05

**Authors:** Haley Peters, Arslan Iqbal, Emily Miller, Sana Khalid, Omar Rahman

**Affiliations:** 1 Critical Care Pharmacy, Indiana University Health, Indianapolis, USA; 2 Critical Care Medicine, Indiana University School of Medicine, Indianapolis, USA; 3 Department of Pharmacy, Indiana University Health, Indianapolis, USA; 4 Critical Care, Indiana University Health, Indianapolis, USA; 5 Pulmonary & Critical Care, Indiana University School of Medicine, Indianapolis, USA

**Keywords:** icu patients, outcomes, clostridium difficile associated diarrhea, continuous enteral vancomycin, clostridium difficile infection

## Abstract

Background and objective

Severe *Clostridium difficile (C. difficile)* infection (CDI)-related colitis is associated with high morbidity and mortality. Current guidelines recommend oral vancomycin plus intravenous metronidazole as the first-line treatment and early total colectomy in case of medication failure. In critically ill patients at high surgical risk and with multiple comorbidities, loop ileostomy creation and enteral vancomycin infusion have been employed albeit with limited success. We hypothesized that continuous enteral vancomycin (CEV) infusion via a postpyloric feeding tube would provide a less invasive, efficacious, and safer route to treat high surgical risk patients.

Methods

All adult (>18 years) non-pregnant patients admitted to the ICU for severe CDI from October 2012 to October 2016 and received CEV after the failure of conventional therapy were included. Vancomycin was prepared as a 1-2-mg/ml enteral solution and run continuously through a feeding pump at 42 ml/hour via a post-pyloric feeding tube. The primary efficacy endpoint was clinical improvement defined as (a) decrease in stool output, (b) decreased vasopressor requirement, or (c) improved leukocytosis, and the secondary endpoint was treatment failure defined as the need for total colectomy or death due to severe CDI.

Results

Our cohort comprised 11 patients in total. The median age of the participants was 64 years, and there were more females (67%) than males (36%). Clinical improvement was seen in seven patients (63%); treatment failure documented as the need for total colectomy was observed in two patients (18%), and death attributable to CDI occurred in three patients (27%).

Conclusion

CEV resulted in clinical improvement in most patients with severe CDI who were at high surgical risk. Sustained intestinal vancomycin delivery may increase luminal concentration and bactericidal effect. The use of a feeding tube and pump provides an effective and less invasive route of vancomycin delivery in critically ill patients.

## Introduction

*Clostridium difficile (C. difficile)* is a Gram-positive anaerobic bacteria and is the leading cause of diarrhea in hospitalized patients [1]. *C. difficile* infection (CDI) is a significant healthcare-associated infection, which is associated with a considerable economic burden throughout the world. In the United States alone, approximately 453,000 cases of CDI are diagnosed and 29,000 associated deaths are reported every year, translating into an economic burden ranging from $436 million to three billion dollars. The rising incidence and severity of CDI could be attributed to the increased use of broad-spectrum antibiotics, patients with comorbid conditions, and the emergence of hypervirulent *C. difficile* strain. The increase in the prevalence of fulminant *C. difficile* colitis has reached alarming proportions in the past few decades [2-4]. As CDI is associated with significant morbidity and mortality, a high degree of suspicion of infection is required in susceptible patient populations for early diagnosis and treatment. CDI commonly presents as diarrhea; however, it ranges from a case of mild diarrhea to the patients having fulminant colitis or small bowel enteritis and recurrent CDIs [5]. Laboratory diagnosis of *C. difficile* requires the detection of *C. difficile-*associated toxin production, which allows for prompt treatment and implementing sufficient precautions so that the spread of further nosocomial infections could be avoided [6].

There are several methods readily available for the diagnosis of CDI in laboratories. The culturing of stool for isolating the *C.difficile* and then applying cytotoxin assay on the isolate is regarded as the gold standard method [7]. However, this method is labor-intensive, subjective, time-consuming, and hence not widely used in the clinical setting. Other diagnostic tests including enzyme immunoassays (EIAs) and polymerase chain reaction (PCR)-based molecular assays have shown promising results [8]. The treatment of choice for CDI continues to be antibiotic therapy. Although antibiotics including metronidazole, fidaxomicin, and vancomycin have been described to be quite effective for this condition [9,10], recent studies that isolated the *C. difficile* suggest reduced susceptibility and increased resistance to these antibiotics, which raises a serious concern in terms of continuing the usage of these antibiotics for the treatment of CDI [11]. Hence, developing new antibiotics and alternative treatment strategies along with devising novel approaches in diagnosing CDI have become significantly important. In this study, we present a case series and report the clinical outcomes in 11 patients admitted to the ICU and suffering from severe CDI, who were treated with continuous enteral vancomycin (CEV) as the main therapy after treatment failure with conventional oral vancomycin treatment.

## Materials and methods

This study was conducted at the Methodist Hospital, an 802-bed Indiana University-affiliated tertiary-care academic medical center. Patients were identified using electronic medical records. A patient who received CEV therapy was defined as a case patient for *C. difficile* colitis. Medical records were evaluated to confirm that the selected patients were diagnosed with *C. difficile* colitis and received CEV therapy for CDI. Data was gathered retrospectively and included demographic characteristics, past medical history, history of surgery, and radiographic information. All adult (>18 years), non-pregnant patients admitted to the ICU for severe CDI between October 2012 and October 2016 who received CEV were included. The decision regarding the initiation of the CEV regimen was made by the respective physician and was started in patients who were poor surgical candidates and had failed the initial conventional treatment. After describing the benefits and side effects of the treatment, verbal consent was obtained from the patient or the legally authorized representative. Continuous treatment included a 1-2-mg/ml enteral solution of vancomycin, which was run continuously through a feeding pump at 42 ml/hour via a post-pyloric feeding tube.

The treatment outcomes were as follows: clinical improvement (decrease in stool output, decreased vasopressor requirement, or improved leukocytosis, survival) and complications such as bowel perforation, readmission to the hospital in case of a recurrent CDI within 30 days after the completion of the treatment for infection, need for surgery, and death due to severe CDI.

## Results

The median age of the patients was 64.8 years, and the majority of them were females [7/11 (63%)]; there were four (36%) males in the cohort, as shown in Table 1.

**Table 1:** Demographics and comorbid conditions of patients admitted to ICU DM: diabetes mellitus; HTN: hypertension; CAD: coronary artery disease; CVA: cerebrovascular accident; AKI: acute kidney injury; CKD/ESRD: chronic kidney disease/end-stage renal disease

Demographics			Comorbidities; 0=no, 1=yes							
Patient	Age, years	Sex; 0=male, 1=female	DM	HTN	CAD	CVA	AKI	CKD/ESRD	Malignancy	Immune suppression
1	80	0	0	0	1	0	1	1	1	0
2	73	0	0	1	1	0	0	0	0	0
3	86	1	0	0	0	0	1	0	0	1
4	65	0	0	0	0	0	1	0	0	0
5	83	1	1	1	0	0	1	1	0	0
6	50	1	1	0	0	0	0	1	0	0
7	86	1	0	1	0	0	1	1	0	0
8	34	0	0	0	0	0	0	0	0	0
9	44	1	1	1	0	0	0	1	0	0
10	76	1	0	1	0	0	1	1	0	0
11	36	1	0	0	0	1	0	0	0	0

The medical histories of the patients included diabetes [n=3, (27%)], hypertension [n=6, (54%)], coronary artery disease [n=2, (18%)], cerebrovascular accident [n=1, (9%)], acute kidney injury [n=6, (54%)], chronic kidney disease/end-stage renal disease [n=6, (54%)], and malignancy [n=1, (9%)]. Only one patient was found to have malignancy located intra-abdominally. Furthermore, among those treated with vancomycin, only one patient was immunosuppressed. During the initial management of CDI, 10/11 patients were treated with oral vancomycin with a minimum dose of 125 mg and a maximum dose of 500 mg with an average duration of 101 hours, as shown in Table 2.

**Table 2:** Duration of oral vancomycin therapy and clinical condition of every patient before starting continuous enteral vancomycin 0=No. 1=Yes WBC: white blood cells; N/A: not available

Patient	Conventional Rx	Duration of conventional Rx (hours)	PO vancomycin dose, mg	WBC, x 10^9^/L	Shock	Lactic acidosis	Toxic megacolon	Ileus	Surgical contraindication
1	1	48	250	22.3	1	0	0	0	0
2	1	48	250	28.4	0	0	0	1	0
3	1	144	250	29.6	1	0	1	0	0
4	1	192	500	15.6	1	0	0	0	1
5	0	N/A	N/A	47.6	1	1	0	0	0
6	1	168	250	60.5	0	1	0	1	0
7	1	N/A	N/A	11.3	1	1	0	0	0
8	1	48	250	24.4	1	0	0	1	0
9	1	60	125	35.7	1	1	0	0	1
10	1	Unknown	250	31.5	1	0	0	0	0
11	1	Unknown	500, 250	33.7	1	1	0	0	0

The average WBC count measured during the initial treatment was 31 x 10^9^/L. Of all the patients who were initially treated with conventional oral vancomycin, nine (81%) experienced shock, four (45%) developed lactic acidosis, one (9%) had toxic megacolon, and three (27%) had ileus; and of these patients, two (18%) were not candidates for surgery. After the treatment failure with conventional oral vancomycin, these patients were transitioned to CEV. Each patient was given enteral vancomycin for an average duration of 168 hours, and the outcomes of this novel treatment are shown in Table 3.

**Table 3:** Duration of continuous enteral vancomycin treatment and clinical outcomes *Perforation was diagnosed on radiologic imaging 0=No. 1=Yes *C.diff: Clostridium difficile*; LOS: length of stay

Patient	Duration (hours)	Clinical improvement	Surgery (tx failure)	Perforation*	Death (2/2, *C.diff)*	LOS (days)	Readmission to ICU	Readmission to hospital	Death at 28 days
1	192	1	0	0	0	16.8	0	1	0
2	360	1	1	0	0	23.9	0	0	0
3	264	1	0	0	0	33	1	0	1
4	384	1	0	1	0	57.3	0	0	0
5	120	1	0	0	0	15.9	0	0	1
6	48	1	0	0	0		0	1	0
7	48	0	0	0	1	3.1	0	0	1
8	24	0	1	1	1	5.1	0	0	1
9	7	0	0	0	1	3.4	0	0	1
10	336	1	0	0	0	23	0	0	0
11	72	0	1	0	0	25.4	0	0	0

Of these 11 patients, seven (63%) showed signs of improvement. Three (27%) patients underwent surgical intervention. Only three (27%) out of the 11 patients succumbed to death secondary to CDI; the additional two deaths were unrelated as either the patients had “do not resuscitate” (DNR) status or were shifted to comfort care after discussion with the patients or family members. The average length of stay (LOS) was 21 days with only one (9%) patient readmitted to the ICU and two (18%) readmitted to the hospital. At 28 days, five (45%) deaths were recorded.

## Discussion

A case of *C. difficile*-associated diarrhea (CDAD)-like disease process was initially reported in 1892 in a 22-year-old female patient treated for the resection of the tumor in the gastric pylorus by Dr. William Osler at the Johns Hopkins University [12]. During the postoperative period, she developed diarrhea, resulting in her death on the 15th postoperative day. The postmortem report showed a pseudomembranous “diphtheritic membrane” seen in the small bowel, which, on cytological examination, showed key inflammatory features of CDAD. After the introduction of antibiotics in the late 1940s and early 1950s, case reports on pseudomembranous enterocolitis became more frequent [13,14]. In the early 1970s, an evolutionary understanding of pseudomembranous colitis emerged after the introduction of the anti-microbial agent clindamycin by Upjohn. In 1974, Tedesco published a study that discussed the association between the drug clindamycin and severe diarrhea in multiple patients after receiving treatment for anaerobic infections [15]. The researchers in the United Kingdom and at the University of Michigan have described the toxigenic nature of pseudomembranous colitis [16]. Eventually, several investigators were able to isolate *C. difficile* from the stool of patients with pseudomembranous colitis [17,18]. CDI can range from diarrhea (usually watery but can be bloody in fulminant cases) to toxic megacolon and severe systemic sepsis. It can be fatal as well due to septic shock and multiorgan failure. The risk factors that can lead to fulminant disease include old age, immunosuppressive therapy, autoimmune disease, and AIDS.

Clinical history and laboratory tests play an important role in the diagnosis of CDI. Consideration of risk factors such as recent antibiotic use, exposure to hospital environments and high-risk patients, and diarrhea for over three days without an identified causative agent is an important prerequisite for the submission of stool for diagnosis. The laboratory-related diagnosis of CDI is still evolving but can include bacterial culture and detection of bacterial toxins or toxin genes from the stool. The most commonly used laboratory methods include toxigenic culture (TC), EIAs for toxins (TcdA or TcdB) or common antigen [glutamate dehydrogenase (GDH)], and real-time PCR [19,20].

The treatment and management of CDI can vary and are based on the clinical presentation, the severity of the illness, and the number of recurrences. Metronidazole and oral vancomycin have been the mainstay of treatment for many years. In addition to these drugs, the new treatment options include novel drugs such as fidaxomicin and nitazoxanide, as well as fecal microbiota transplantation [21]. The use of probiotics either in the treatment or prevention of CDI to restore colonic microflora has been the topic of study in numerous articles. However, the Society for Healthcare Epidemiology of America/Infectious Diseases Society of America (SHEA/IDSA) guidelines do not recommend the use of probiotics in CDI due to limited data and concern that the potential risk of bloodstream infection may outweigh the benefits [22]. Vancomycin was the first drug approved by the FDA for the treatment of CDI. SHEA/IDSA guidelines recommend the use of oral vancomycin for severe CDI and a combination of vancomycin and metronidazole for severe-complicated infection [10], although response rates in mild to moderate disease are similar for both metronidazole and vancomycin. However, the preferred treatment is vancomycin in severely ill patients due to higher cure rates in these patients (97% versus 76%), although there were no significant differences in the subsequent relapse rates between these two treatments [23].

Mizumura et al. have studied continuous vancomycin administration through a long intestinal tube as a treatment option for CDI. They presented a case of a 76-year-old male patient who had been previously healthy but experienced severe diarrhea (8,000 mL per day) and was diagnosed with CDI after postoperative laparotomy for small bowel obstruction. He was successfully treated with CEV through a long intestinal tube placed in the terminal ileum. This method ensured the reliable delivery of vancomycin to the colon [24]. In our study, we used CEV as a treatment for patients with severe CDI admitted to ICU. All patients were started on CEV after they failed to respond to conventional oral vancomycin. At the time of starting CEV, 80% of the patients were in shock and 20% had been deemed unfit for surgery due to their very frail condition. Post-pyloric continuous vancomycin was given through a feeding pump for an average duration of 168 hours for all patients and improvement was seen in seven (68%) of them.

Our study was limited by its retrospective design, and we also did not confirm the eradication of the production of cytotoxin for each patient. Although some complications like perforation, the need for surgical intervention, and death occurred in a few patients, we were unable to determine the attributable risk associated with CEV from these observational data. In addition, because of the retrospective nature of the data, we failed to observe issues related to the efficacy, toxicity, and safety of the therapy.

## Conclusions

Based on our observations, high clinical response rate, and favorable outcomes, CEV can be used as a treatment option in patients with severe CDI who are not responsive to conventional oral vancomycin treatment. However, further prospective studies are required to gain more insights into the significance of CEV treatment.

## References

[1] Garey KW, Jiang ZD, Yadav Y, Mullins B, Wong K, Dupont HL (2008). Peripartum Clostridium difficile infection: case series and review of the literature. Am J Obstet Gynecol.

[2] Peng Z, Ling L, Stratton CW, Li C, Polage CR, Wu B, Tang YW (2018). Advances in the diagnosis and treatment of Clostridium difficile infections. Emerg Microbes Infect.

[3] Shin JH, Chaves-Olarte E, Warren CA (2016). Clostridium difficile infection. Microbiol Spectr.

[4] de Curraize C, Rousseau C, Corvec S (2018). Variable spectrum of disease and risk factors of peripartum Clostridium difficile infection: report of 14 cases from French hospitals and literature review. Eur J Clin Microbiol Infect Dis.

[5] Napolitano LM, Edmiston CE Jr (2017). Clostridium difficile disease: Diagnosis, pathogenesis, and treatment update. Surgery.

[6] Kelly CP, LaMont JT (2008). Clostridium difficile--more difficult than ever. N Engl J Med.

[7] Reller ME, Lema CA, Perl TM, Cai M, Ross TL, Speck KA, Carroll KC (2007). Yield of stool culture with isolate toxin testing versus a two-step algorithm including stool toxin testing for detection of toxigenic Clostridium difficile. J Clin Microbiol.

[8] Ryder AB, Huang Y, Li H (2010). Assessment of Clostridium difficile infections by quantitative detection of tcdB toxin by use of a real-time cell analysis system. J Clin Microbiol.

[9] Leffler DA, Lamont JT (2015). Clostridium difficile infection. N Engl J Med.

[10] Cohen SH, Gerding DN, Johnson S (2015). Clinical practice guidelines for Clostridium difficile infection in adults. Cambridge Core.

[11] Spigaglia P (2016). Recent advances in the understanding of antibiotic resistance in Clostridium difficile infection. Ther Adv Infect Dis.

[12] (1986). Classic articles in colonic and rectal surgery. John Miller Turpin Finney 1863-1942. Gastro-enterostomy for cicatrizing ulcer of the pylorus. Dis Colon Rectum.

[13] Hummel RP, Altemeier WA, Hill EO (1964). Iatrogenic Staphylococcal enterocolitis. Ann Surg.

[14] Wakefield RD, Sommers SC (1953). Fatal membranous staphylococcal enteritis in surgical patients. Ann Surg.

[15] Tedesco FJ (1976). Clindamycin-associated colitis. Review of the clinical spectrum of 47 cases. Am J Dig Dis.

[16] Larson HE, Price AB (1977). Pseudomembranous colitis: presence of clostridial toxin. Lancet.

[17] George W L, Sutter V, Finegold S (1978). Toxigenicity and antimicrobial susceptibility of Clostridium difficile, a cause of antimicrobial agent-associated colitis. Curr Microbiol.

[18] Larson HE, Price AB, Honour P, Borriello SP (1978). Clostridium difficile and the aetiology of pseudomembranous colitis. Lancet.

[19] Deshpande A, Pasupuleti V, Rolston DD, Jain A, Deshpande N, Pant C, Hernandez AV (2011). Diagnostic accuracy of real-time polymerase chain reaction in detection of Clostridium difficile in the stool samples of patients with suspected Clostridium difficile Infection: a meta-analysis. Clin Infect Dis.

[20] Badger VO, Ledeboer NA, Graham MB, Edmiston CE Jr (2012). Clostridium difficile: epidemiology, pathogenesis, management, and prevention of a recalcitrant healthcare-associated pathogen. JPEN J Parenter Enteral Nutr.

[21] Khanna S, Pardi DS (2014). Clostridium difficile infection: management strategies for a difficult disease. Therap Adv Gastroenterol.

[22] Pillai A, Nelson R (2008). Probiotics for treatment of Clostridium difficile-associated colitis in adults. Cochrane Database Syst Rev.

[23] Zar FA, Bakkanagari SR, Moorthi KM, Davis MB (2007). A comparison of vancomycin and metronidazole for the treatment of Clostridium difficile-associated diarrhea, stratified by disease severity. Clin Infect Dis.

[24] Mizumura N, Demura K, Kawasaki M (2015). Continuous administration of vancomycin through a long intestinal tube for Clostridium difficile infection. Intern Med.

